# Viral diversity is linked to bacterial community composition in alpine stream biofilms

**DOI:** 10.1038/s43705-022-00112-9

**Published:** 2022-03-30

**Authors:** Meriem Bekliz, Paraskevi Pramateftaki, Tom Jan Battin, Hannes Peter

**Affiliations:** River Ecosystems Laboratory, Ecole Polytechnique Fédérale de Lausanne, CH-1015 Lausanne, Switzerland

**Keywords:** Microbial ecology, Bacteriophages

## Abstract

Biofilms play pivotal roles in fluvial ecosystems, yet virtually nothing is known about viruses in these communities. Leveraging an optimized sample-to-sequence pipeline, we studied the spatiotemporal turnover of dsDNA viruses associated with stream biofilms and found an astounding diversity to be structured by seasons and along the longitudinal gradient in the stream. While some vOTUs were region- or season-specific, we also identified a large group of permanent biofilm phages, taxonomically dominated by Myoviridae. Comparison of the observed viral distribution with predictions based on neutral community assembly indicated that chance and dispersal may be important for structuring stream biofilm viral communities. Deviation from neutral model predictions suggests that certain phages distribute efficiently across distant locations within the stream network. This dispersal capacity appears to be linked to EPS depolymerases that enable phages to efficiently overcome the biofilm barrier. Other phages, particularly vOTUs classified as Siphoviridae, appear locally overrepresented and to rely on a lysogenic life cycle, potentially to exploit the spatial distribution of bacterial populations in stream biofilms. Overall, biofilm viral and bacterial community turnover were significantly coupled. Yet, viral communities were linked to the presence of the most abundant bacterial community members. With this work, we provide a foundational ecological perspective on factors that structure viral diversity in stream biofilms and identify potentially important viral traits related to the biofilm mode of life.

## Introduction

Many microorganisms are adapted to life in surface-attached biofilms, which may have been the predominant microbial lifestyle for more than 3 billion years [1, 2]. Biofilms also dominate microbial life in streams and rivers, where members of all domains of life form complex and dynamic communities [3]. Stream biofilm communities are diverse, fulfill pivotal ecosystem processes and contribute to global element fluxes. Yet, the ecological role of viruses and particularly of bacteriophages in stream biofilm communities remains unknown. This gap in our knowledge can mainly be attributed to the lack of marker genes for molecular analyses and technical challenges related to the study of the minuscule viral particles in the biofilm matrix.

As obligate intracellular parasites, viruses have been detected in virtually all habitats on Earth [4, 5]. Research on viral ecology has been pioneered in marine pelagic habitats, where bacteriophages regulate bacterial mortality, community composition, and diversity. Work on the virosphere of other microbiomes [6], including soils [7], microbial mats [8], and the human gut [9], has further highlighted the role viruses play in shaping bacterial community ecology and evolution through lysis-related selection, host-cell reprogramming, and horizontal gene transfer [5]. In fluvial ecosystems, our understanding of viral communities, besides recognition of the potential to impact global biogeochemical cycles [10], remains limited to the variable nature of suspended and particle-associated viral-like particle (VLP) abundance [11]. And while the biofilm matrix may represent a physical barrier to phages [12], the abundance of both phages and biofilms in streams suggests that phages may also play important roles in stream biofilms [3]. Mainly based on computational simulations of single-species bacterial cultures of medical importance [13, 14], it appears that the biofilm lifestyle may confer protection against phage infection by inhibiting phage transport by matrix extend, composition, and architecture [15]. At the same time, phages trapped in the biofilm matrix may remain active and can eliminate newly arriving cells [12], potentially providing benefits to biofilm-dwelling bacteria. Other mutualistic biofilm–phage interactions have also been reported, particularly when temperate phages introduce auxiliary metabolic genes or when the presence of phages in the biofilm matrix provides structural benefits [16]. Therefore, given the shared evolutionary history and frequent phage–biofilm encounters [17], it is reasonable to assume that phages also play a pivotal role in shaping biofilm communities in natural environments, such as in streams and rivers.

With the advent of viral metagenomics and the development of sophisticated procedures to extract and purify viral particles from the biofilm matrix, we now have the tools to explore phage diversity in stream biofilms [18]. Here, we studied the spatial and temporal turnover of viral communities associated with benthic stream biofilms in an alpine catchment. Understanding the processes that generate patterns in diversity, composition, and turnover is a critical first step toward appreciating the role of viruses in shaping microbial communities. To achieve this, we obtained viromes of benthic biofilms from ten sites along an altitudinal gradient in a mountain stream sampled during four seasons (*Methods*). Given the heterogeneous and dynamic structure of stream benthic biofilm communities, we expected to discover a large viral diversity and pronounced turnover. We hypothesized that either microbial community patchiness at the local scale (i.e., length scale of meters) or larger scale spatial patterns that emerge as the stream moves through the alpine landscape (i.e., length scale of kilometers) dictate viral community composition. We further speculated that specialized phages may benefit from certain traits such as depolymerases to access cells embedded in an extracellular polymeric matrix, and, in combination with lytic infections, that this allows phages to disperse efficiently across distant sites in a stream ecosystem. On the other hand, analogous to the density-dependence of viral lifestyles in pelagic ecosystems, particular viruses may favor a lysogenic lifestyle driven by densely packed microbial populations in biofilms. We expect such a phenomenon to be reflected in disproportionately high local abundances and limited spread across more distant sites within the fluvial ecosystem. To resolve such assembly patterns, we compared observed distribution patterns with predictions based on neutral theory of ecology [19]. This sampling-based model assumes neutrality (i.e., equality) in terms of fitness (i.e., growth, death and dispersal rate) among viral taxonomic units (vOTUs) and predicts occurrence frequency from abundance across the metacommunity. Model prediction thus suggests that abundant vOTUs are more widely spread across the metacommunity, a phenomenon linked to dispersal. Finally, we hypothesized that the spatiotemporal turnover in the viral community is linked to the bacterial biofilm community. With this work, we provide a foundational ecological perspective on the role viruses play in stream biofilms.

## Methods

### Sampling

We conducted sampling campaigns in spring (April 2018), summer (July 2018), autumn (October 2018), and winter (February 2019) in the Vièze in Switzerland. Downstream distances were obtained from digital elevation models (25 × 25 m spatial resolution, swisstopo) processed using the SSN [20] package in R. At each site, stream biofilm slurries were collected from stones (5–15 cm in diameter, average sampled surface area: 0.86 ± 0.25 m^2^) using sterile brushes. Slurries were kept on ice until processing. Physical–chemical parameters including streamwater temperature, pH, oxygen, and specific conductivity were measured using a multiparameter probe (WTW, Xylem Inc., USA). Samples for the analysis of dissolved organic carbon were filtered on through GF/F filters (Whatman, USA) into pre-combusted glass vials and analyzed on a TOC analyzer (Sievers M5310c, GE Analytical Instruments, USA). Ammonium (NH_4_) was measured using an improved fluorometric method (detection limit >50 μg/L) [21]; soluble reactive phosphorus was measured using the spectrophotometric molybdovanadate method and major anions and cations were measured using ion chromatography (ICS‐3000 Dionex, USA).

Viral-like particles were extracted and purified from biofilm slurries using a previously described optimized procedure [18]. Briefly, we centrifuged biofilm slurries at 100 g (15 min, 4 °C) (5810R, Eppendorf, Germany) to remove large inorganic particles and concentrate the supernatant using a medium-scale tangential flow filtration system equipped with a 100 kDa filter (GE Healthcare, USA) [22]. Following the recommendation of Danovaro and Middelboe [23], we used sonication in combination with tetrasodium pyrophosphate to separate viruses from biofilms. Then, samples were centrifuged (4 °C) at 3234 *g* for 15 min and the supernatant was sequentially filtered through 0.8 μm and 0.45 μm filters (Whatman, USA), respectively to separate viral particles from debris and microbial cells. To eliminate contaminating extracellular nucleic acids, DNase I at a final concentration of 2.5 U/μL (Life Technologies, Germany) was used. To confirm the removal of contaminant DNA, PCR targeting the 16S rRNA gene was performed using the universal primer pair 341f (5′-CCTACGGGNGGCWGCAG-3′) and 785r (5′-GACTACHVGGGTATCTAAKCC-3′). Finally, VLPs were purified using sucrose density gradient ultracentrifugation [18].

### Viral-like particle and bacteria enumeration

We enumerated VLPs using epifluorescence microscopy (AxioImager Z2, Zeiss, Germany) [24]. For this, samples of extracted and purified VLPs were fixed with formaldehyde, stained with SYBR Gold (Molecular Probes, ThermoScientific, USA), and incubated at room temperature in the dark (30 min). After incubation, each sample was filtered onto a 0.02 μm pore size membrane filter (Anodisc, Whatman). The filters were mounted on glass slides with a drop of VectaShield mounting medium (Vector Laboratories, USA). VLPs were visualized under 488 nm excitation and 512 nm emission. For each sample, 20 randomly selected images were acquired with a camera (Axiocam 506 mono, Zeiss) mounted onto the microscope. VLPs were discriminated from bacteria by size (0.015–0.2  μm) and enumerated using a custom script in Fiji [25]. For bacterial cell counting, untreated biofilm slurries were fixed with 3.7% formaldehyde (final concentration). Bacterial cells were disintegrated from the biofilm matrix using 0.25 mM tetrasodium pyrophosphate in combination with rigorous shaking (1 h) and sonication (Bransonic Sonifier 450, Branson) on ice (1 min). Cells were stained using Syto13 (15 min, room temperature) and counted on a flow cytometer (NovoCyte, ACEA Biosciences).

### Sequencing and bioinformatics

To generate metagenome sequencing libraries, we extracted viral DNA [18] and constructed sequencing libraries (Nextera DNA Flex) using 1 ng of input DNA. Paired-end sequencing (2 × 300 bp) was performed on a MiSeq System (Illumina, USA) at the Lausanne Genomic Technologies Facilities. Raw sequence datasets have been submitted to the European Nucleotide Archive under accession number PRJEB50322.

Bioinformatic processing was performed as described previously [18]. Briefly, sequences were quality trimmed to remove sequencing adapters, short and low-quality reads and reads matching the human reference genome using bbduk, bbmap and tadpole [26]. Reads were then de novo assembled using SPADes [27] in single-cell mode and kmers set to 21, 33, 55, 77, 99, and 127. Putative viral contigs were then identified using Virsorter [28] (category 1–2) and DeepVirFinder [29] (FDR adjusted *p* value < 0.01). Subsequently, viral contigs were clustered into vOTUs [30] based on 95% average nucleotide identity over 85% of the shorter sequence (MUMmer 3.1) [31]. Finally, reads were mapped to representative vOTU sequences (i.e., largest contig) using BWA-MEM [32] (default settings) and samtools [33] and normalized to RPKM mapped reads. Taxonomy was assigned to representative vOTUs sequences using blastx (*E* value < 10^−6^, identical matches >30% and bitscore >200) against a concatenated database including NCBI viral reference genomes and IMG/VR v3 [34] sequences. Taxonomic composition was then visualized using metacoder [35] in R. Viral life cycle predictions (lysogenic/lytic) were obtained using BACPHLIP [36] with default settings. BACPHLIP is a random forest classifier which allows identifying lysogeny-associated protein domains. Since robust classification requires complete genomes, we restricted our analysis to 256 vOTU contigs classified as complete (100%) by checkv (including categories “complete” and “high quality”). Viral depolymerases in our dataset were identified by blastx searches (*E* value < 10^−6^, bitscore:qlength >0.7 and pident >0.4) against 202 previously published depolymerases [37–39] (SI Table 2). Bacterial community composition was analyzed from subsamples of biofilm slurry collected onto 0.2 µm filters and frozen at −80 °C. Bacterial DNA was extracted using the DNeasy Power Soil kit (QIAGEN), following manufacturers instructions. Bacterial 16S rRNA genes were then amplified using PCR and the 341f (5′CCTACGGGNGGCWGCAG-3′) and 785r (5′-GACTACHVGGGTATCTAAKCC-3′) primer pair. We prepared sequencing libraries using the Nextera XT kit (Illumina), equimolar pooled and sequenced on a 300 bp paired-end MiSeq (Illumina) at the Lausanne Genomic Technology Facility. Sequencing adapters were clipped from raw reads, which were then denoised and clustered into Amplicon Sequence Variants using DADA2 [40] (vers. 1.14) using QIIME2 [41]. Uni- and multivariate statistical analyses were performed in R, using the package vegan [42]; neutral model simulations were performed using the R package MicEco (https://github.com/Russel88/MicEco).

## Results and discussion

### Viral-like particle abundance

**Fig. 1:**
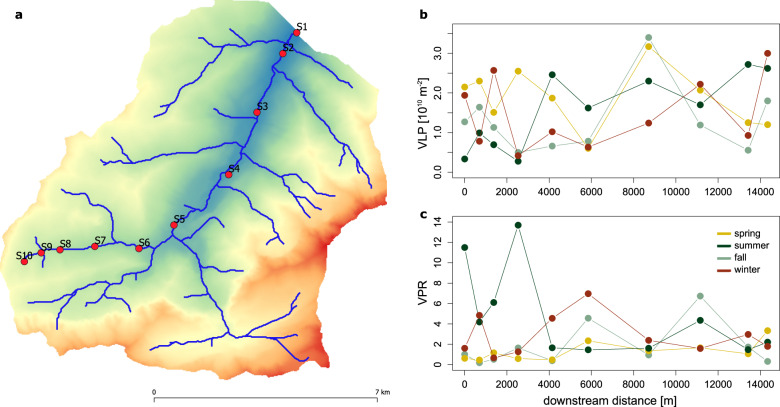
No evidence for a downstream accumulation of VLPs. Viral-like particles (VLP) were purified from 10 sites sampled during four seasons along an altitudinal gradient in an alpine stream (Vièze, Switzerland) (**a**). Neither VLP abundance (**b**) nor Virus-to-Prokaryote Ratios (VPR; (**c**)) showed pronounced spatial or temporal trends.

The 10 sampling sites were equidistantly (average distance: 1.6 km) distributed between 1689 and 717 m above sea level in a 95.7 km^2^, pristine catchment and covered a flow-connected distance of 14.3 km (Fig. 1, *Methods*).

Viral-like particle (VLP) counts normalized to areal coverage of the stream biofilm ranged from 2.8 × 10^9^ to 3.4 × 10^10^ VLP m^−2^. On average, VLP abundance was highest in summer with 1.87 ± 0.75 × 10^10^ VLP m^−2^; however, there were no statistically significant seasonal differences in VLP abundance (repeated-measures ANOVA, *F* = 0.87, *p* = 0.47). VLP numbers did not exhibit a continuous spatial tendency, except during fall when VLP numbers increased significantly with downstream distance (*r* = 0.81, *p* < 0.01). This was strikingly different from the distribution of prokaryotic cells in biofilms as estimated by flow cytometry, which, across all seasons, tended to increase with increasing downstream distance (*r* = 0.36, *p* = 0.02). Consequently, VLPs and prokaryotic cell counts were not significantly correlated (*r* = 0.16, *p* = 0.32) and virus-to-prokaryote ratios (VPR) ranged from 0.2 to 13.7, with an overall mean of 2.7. While being generally low compared to pelagic ecosystems in which VPR typically vary around 10, VPR in stream biofilms exhibited significant seasonal differences, being highest in autumn and winter and lowest in spring and summer (ANOVA, *F* = 3.36, *p* = 0.03). It should be noted that VLP abundance might be somewhat underestimated given that large viruses (e.g., members of the Nucleocytoviricota) are excluded. However, taxonomically identified putative giant viruses contribute on average only 1.47% to vOTU relative abundance.

The apparent uncoupling between viral and prokaryotic community sizes in stream biofilms may be attributed to lags in population dynamics. In streams, prokaryotic members of benthic biofilms grow rapidly after disturbances as induced by storms, for instance. These dynamics vary predictably between seasons, in dependence of nutrient availability and stream water temperature. In spring, snow melt delivers nutrients to alpine streams, which, together with increasing streamwater temperatures and light availability in summer stimulates growth of benthic biofilms and triggers a peak in prokaryotic cell numbers. During this phase of rapid biofilm growth, viruses appear to lag behind prokaryotic communities, as reflected in low VPR. In autumn and winter, however, when disturbances are less common and stream water temperature is low, viruses may become relatively more abundant in stream biofilms. However, whereas density-dependent viral predation may underly the coupling between phage and bacterial abundance in pelagic ecosystems, the spatial organization of bacterial populations in stream biofilms may impose fundamentally different constraints on phage predation [13, 14]. Strategies that allow phages to overcome the biofilm matrix barrier may thus be more relevant for the ecological success of phages in stream biofilms than density-dependent encounter rates.

### Viral diversity

Employing a recently established sample-to-sequence pipeline [18], we obtained viromes from 39 stream biofilm samples. In total, we obtained 6.07 × 10^7^ reads, of which on average 77.5 ± 12.7% were retained after quality trimming. For each sample, on average 58004 contigs were assembled, of which 318 ± 195 contigs were larger than 5000 bp. We then identified 5667 putative viral contigs larger 5000 bp which clustered into 1445 vOTUs with a median contig size of 11704 bp. CheckV [43] classified 1.40% of these sequences to be complete, 0.61% to high-, 12.80% to medium-, and 73.61% to low-quality sequence categories. With on average 94.7 kb and 208.8 kb, complete and high-quality contigs were larger than medium- (57.5 kb) and low-quality (13.3 kb) contigs.

**Fig. 2:**
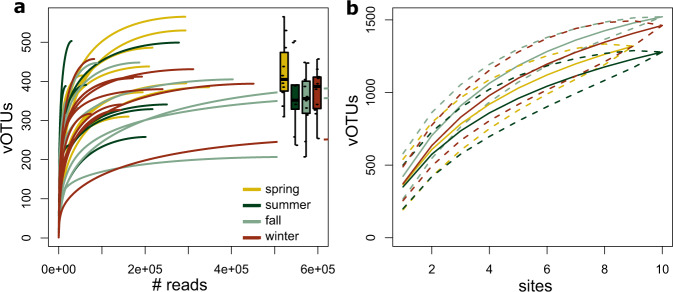
Virome sequencing revealed a large viral Operational Taxonomic Unit (vOTU) richness totaling 1445 vOTUs. Plateauing of individual sample rarefaction curves (**a**) indicates that sequencing effort generally sufficed to describe vOTU diversity, which ranged between 122 and 360 vOTUs. The insert boxplot summarizes vOTU richness across seasons. vOTU accumulation curves for the different seasons also approached an asymptote (**b**), indicating that the spatial sampling effort captured the regional viral biofilm diversity reasonably well, but also that not the entire viral metacommunity was sampled in any of the four seasons.

Individual rarefaction curves saturated (Fig. 2), indicating that our sequencing effort sufficed to characterize biofilm viral diversity. On average, we detected 250 ± 56 vOTUs in our samples, with considerably higher vOTU richness in spring (274 ± 60) than in the other seasons when vOTU richness averaged between 235 and 247 vOTUs. Contrary to our expectation that biofilm viral diversity would increase with downstream distance due to accumulation effects, we did not observe significant longitudinal gradients in vOTU richness. vOTU relative abundance was substantially skewed, with only a few vOTUs dominating viral abundance in each sample. More specifically, the 10 most abundant vOTU in each sample accounted on average for 72.3 ± 16.3% RPKM. This resulted in low viral community evenness, averaging 0.097 ± 0.071. Strikingly, there was substantial spatial and temporal turnover in the identity of these dominant viral community members (*n* = 351). Across all seasons and samples, only 18.2% of the dominant vOTUs were more than once among the top five most abundant viral community members.

**Fig. 3:**
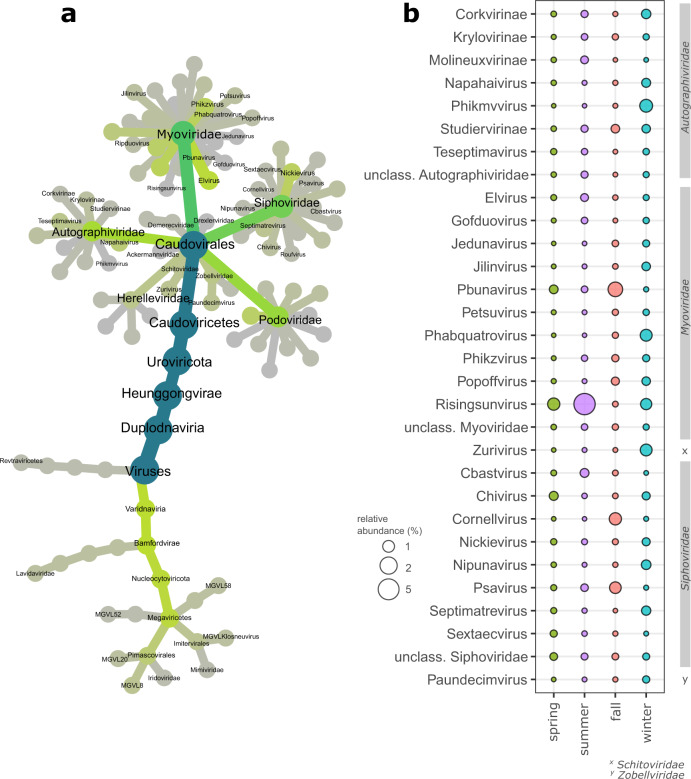
Taxonomic structure of stream biofilm viral communities. Taxonomic classification of vOTUs revealed a large taxonomic diversity of dsDNA viruses found in stream biofilms (**a**). Shown is a taxonomy tree, that, comparable to a phylogenetic tree, depicts the hierarchical structure of viral taxonomies detected in our samples. Nodes reflect taxonomic units, and their color reflects their taxonomic diversity. **b** The most abundant viral taxonomies exhibit pronounced seasonal dynamics.

Taxonomic classification of vOTUs further revealed the massive diversity of dsDNA viruses in stream biofilms. While 59.9% of vOTUs could not be further classified than being a member of Caudovirales Across the entire dataset, we detected 66 different viral genera affiliated to 18 viral families (Fig. 3a). Most stream biofilm dsDNA viral diversity was associated with members of the Caudovirales order and presumably bacteriophages. However, we also detected members of Mimiviridae, giant viruses are known to infect ameba. Among the Caudovirales, members of Myo-, Sipho- and Autographiviridae were abundant and prevalent among stream biofilm samples, but vOTUs classified as Podoviridae, Ackermannviridae, Chaseviridae, Drexlerviridae, and Herelleviridae also contributed to viral diversity. Strikingly, several genera of Myoviridae were particularly abundant across all seasons. For instance, vOTUs classified as Risingsunvirus, Pbunavirus, and Elvirus, were among the most abundant viral community members across all seasons. Typically, however, most viral genera and (sub-) families exhibited pronounced seasonality (Fig. 3b). For instance, abundant genera of Siphoviridae, such as Chivirus, Cornellvirus and Psavirus, and Nonanavirus showed distinct seasonal peaks. The observation of pronounced seasonality, both in terms of taxonomic turnover but also in the replacement of abundant vOTUs in stream biofilm viral communities resembles temporal dynamics observed in the ocean [44], lakes [45], and bogs [46].

**Fig. 4:**
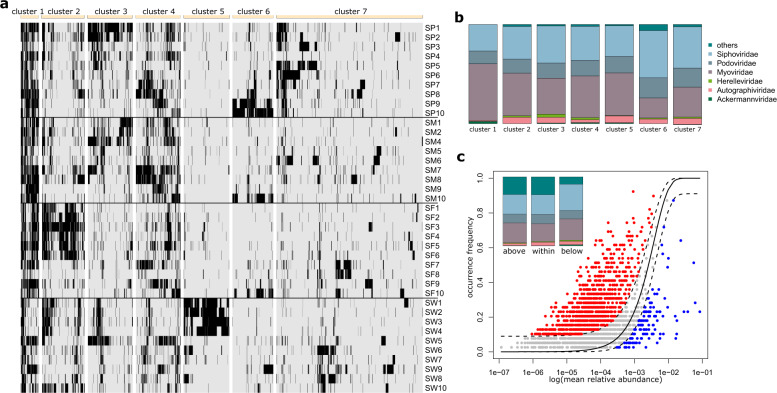
Patterns of prevalence reflect different viral assembly processes. Each bar in the heatmap (**a**) reflects the presence (black) or absence (gray) of an vOTU (column) across all samples (rows). Seven clusters of vOTUs exhibit distinct spatiotemporal patterns (see main text). The barchart (**b**) shows the broad taxonomic composition of classified vOTUs in these seven clusters. Stream biofilm viral communities showed a significant fit to ecological null model prediction (**c**, dotted line show 95% confidence intervals). Approximately half of all vOTUs fell within the null model prediction (gray symbols), but a comparable number of vOTUs was found disproportionately more frequently than expected by chance (red symbols). Only a minor fraction of vOTU diversity was found below the 95% CI of the neutral model prediction (blue symbols). The insert shows taxonomic composition of classified vOTUs in these three categories (same colors as in panel (**b**)).

Besides seasonal preferences, vOTUs also showed pronounced spatial turnover (Fig. 4). Based on agglomerative clustering of presence-absence patterns across sites and seasons, we identified a group of vOTUs that was present across most sites in all seasons (cluster 1, *n* = 107, Fig. 4a). This group of vOTUs was markedly enriched in vOTUs classified as Myoviridiae, accounting for 57.0%, while the relative abundance of Myovirididae across the entire dataset averaged 27.18%. We further observed two distinct clusters of vOTUs that appeared to be season specific. For instance, cluster 5 (*n* = 271), enriched in vOTUs classified as Autographiviridae, was nearly exclusively detected in the four downmost sites in winter, whereas vOTUs in cluster 6 (*n* = 242), enriched in Siphoviridae and Podoviridae occurred predominantly in the most upstream sites in spring. We also identified a group of vOTUs (cluster 3, *n* = 264) that were appeared to be restricted to the uppermost sites in the catchment during spring, summer and winter but absent in fall. W On the other hand, cluster 2 vOTUs were predominantly present in fall. The largest cluster (cluster 7, *n* = 867) showed no clear spatial or temporal distribution patterns. However, the most prevalent and seasonally abundant vOTUs were dominated by Myoviridae, whereas members of Siphoviridae tended to be either site-specific, or show a rather stochastic spatio-temporal distribution. To further quantify these dynamics, we evaluated whether vOTUs adhere to neutral theory of ecology predictions [19]. Neutral theory of ecology predicts, under the assumption of equal ecological fitness among taxa, that stochastic processes, such as random dispersal, govern community assembly. While different assembly processes may be acting in concert, the fit of taxa distribution patterns to neutral model prediction can serve as indirect evidence for the importance of stochastic assembly mechanisms, such as, for instance the role of dispersal of taxa within a metacommunity. The fit to neutral model predictions can be tested by the relationship between occurrence frequency and relative abundance for taxa of the same metacommunity, basically stating that taxa that are abundant in the metacommunity are also likely to be widespread. Comparing the fit of stream biofilm viral communities to this neutral model prediction (Akaike Information Criterion; AIC = −4324) and to binomial distribution models (which would indicate that local viral communities are random subsets of metacommunity in the absence of dispersal and drift) (AIC = 1074), clearly supported the neutral model. Indeed, the majority of vOTUs (50.8%) fell within neutral model prediction intervals (i.e., 95% confidence intervals), indicating that random dispersal is an important community assembly strategy for many stream biofilm viruses. However, we also identified a large number of vOTUs (43.7%) enriched in Myoviridae, that were present in more sites than expected by the neutral model, reflecting vOTUs that are particularly widespread among stream biofilm communities and hence might be particularly well adapted to a biofilm lifestyle. On the other hand, we also identified a few vOTUs (5.5%) that were found less frequently than predicted by the neutral model and enriched in Siphoviridae, indicating a viral community subset with particularly limited dispersal capacities.

These patterns of spatio-temporal turnover of taxonomic groups highlight the various spatial strategies that stream biofilm viral communities might engage in. These findings are thus highly relevant to better understand the ecology of viruses in stream ecosystems, because they indicate that viral communities do not assemble by purely stochastic processes, such as drift from up- to downstream sites. Rather, these patterns reveal fine-scaled spatial dynamics and suggest different viral strategies in stream biofilm metacommunities. For example, viruses infecting biofilm-dwelling bacteria might require depolymerase enzymes to access cells embedded in the extracellular matrix [38, 47] and consequently, such viruses could be particularly widespread among stream biofilm metacommunities. On the other hand, lysogenic, pseudolysogenic and chronic infection life cycles may also offer benefits to phages infecting biofilm-forming bacteria. While depolymerase activity may ease the access of phages to bacteria embedded in a matrix composed of extracellular polymeric substances (EPS), phages embedded in host genomes (or in the case of pseudolysogeny as an extrachromosomal element), may, upon switching to a lytic infection, rather rely on locally high host population densities within biofilms to disperse. However, on the scale of a stream biofilm viral metacommunity, one would expect that the ability to gain access to biofilm bacteria, would result in a wider spread. To substantiate this notion, we compiled a database of 203 putative viral depolymerases and searched for homologs sequences among vOTU (blastx, evalue < 1e-06, bitscore:qlen >0.7 and/or pident >0.4). Indeed, 90 of the 203 putative viral depolymerases showed significant sequence similarity with 198 vOTU sequences (i.e., 6% of the overall vOTU diversity). We were able to obtain taxonomic classification for 80 of these 198 vOTUs, and found that all large Caudovirales families were represented (i.e., Myoviridae, *n* = 31, Siphoviridae, *n* = 17, Podoviridae, *n* = 15, Autographiviridae, *n* = 13, Ackermannviridae, *n* = 2, and Herelleviridae, *n* = 1). This suggests that depolymerase activity may be widespread among viruses infecting bacteria in stream biofilms. Although both the number of potential depolymerases included in our database and the number of classified vOTUs was limited, we observed that depolymerase-harboring Myoviridae vOTUs corresponded the expectation based on the overall relative abundance of Myoviridae, pointing toward the importance of dispersal for this important viral family. Siphoviridae, in contrast, were relatively underrepresented among depolymerase-harboring vOTUs. In combination with neutral model predictions, this may point towards a fundamental difference between Siphoviridae and Myoviridae in infecting stream biofilm bacteria. While Myoviridae may rather rely on efficiently spreading across distant biofilm patches facilitated by an ability to decompose the EPS matrix, many members of Siphoviridae seem to lack this ability.

To investigate our second hypothesis, that lysogeny might be a successful viral life cycle strategy to spread locally within biofilm patches, we used BACPHLIP [36]. BACPHLIP predicted with high probability (>75%) a lysogenic life cycle for 58 out of 256 complete viral genomes and a lytic life cycle for 177 viral genomes. For the remaining 21 complete viral genomes in our dataset, BACPHLIP did not result in sufficiently high prediction probability (i.e., <75%). Across the entire dataset, these lytic phages accounted for 25.8% of mean viral relative abundance, whereas lysogenic phages accounted for only 7.6% of mean viral relative abundance. For the majority of 66.6% of viral relative abundance, however, we could not assign a viral lifestyle. Yet, we observed a moderate seasonal switch in the contribution of lytic and lysogenic phages to community abundance. While in spring and summer lysogenic phages accounted on average for 13.1% of relative abundance, their contribution dropped to 2.0 and 2.8% in fall and winter, respectively. Concomitantly, the average contribution of lytic phages to community abundance increased markedly from 21.2 and 17.0% in spring and summer to 32.2 and 31.9% in fall and winter. This is a striking observation, given that VPR, driven by seasonal changes in bacterial abundance, were low in spring and summer and high in fall and winter. This suggests that similar to pelagic ecosystems, host density also plays an important role in stream biofilm communities by favoring lytic replication.

Linking viral life cycles to spatial distribution patterns, we found that the majority (56.5%) of identified lytic and lysogenic phages fell within the neutral model prediction confidence intervals, whereas 20.4% of these phages were found above and the remaining 23.1% below the neutral model prediction. Contrary to our expectation that lysogenic phages might be less common than expected by chance across the viral metacommunity (i.e., below the neutral model prediction), both lytic and lysogenic phages were found in comparable fractions above (lytic: 20.5%, lysogenic: 19.3%) and below (lytic: 25.2%, lysogenic: 14.1%) neutral model prediction. Resolving the taxonomies of these phages, however, we found that Myoviridae (30.5%) and Siphoviridae (33.7%) contributed similar to lytic viral diversity, whereas Myoviridae (7.4%) were clearly underrepresented among lysogenic phages, which were dominated by phages classified as Siphoviridae (47.2%) and Podoviridae (18.3%). Taken together, this supports the notion that phages common in stream biofilms may follow different life cycle strategies. Although this is clearly at a coarse taxonomic resolution, it appears that Myoviridae may rather use depolymerases to gain access to biofilm-dwelling bacteria, fostering their dispersal across distant sites in a stream network. Siphoviridae and Podoviridae, on the other hand, appear to rather rely on lysogenic replication to eventually infect localized bacterial populations embedded in the biofilm matrix. Future research, ideally involving targeted experimentation, may resolve these suggested fundamental differences among phages specialized on biofilm communities.

### Coupling between viral and bacterial stream biofilm communities

**Fig. 5:**
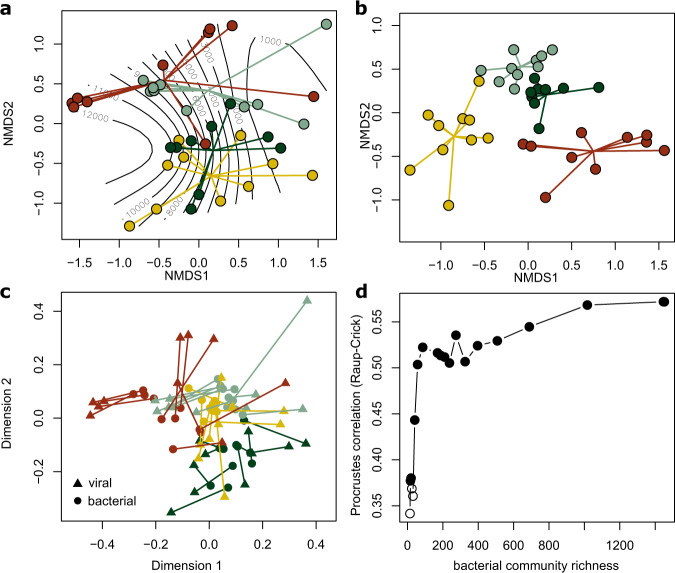
Viral and bacterial community composition are linked. Unconstrained ordination (non-metric multidimensional scaling), **a** shows the compositional turnover of stream biofilm viral communities across sites and among seasons (colors same as in Fig. 1). The lines in the background indicate the downstream distance of each site. **b** It provides the same ordination for bacterial communities characterized by 16S rRNA gene sequencing. Note the more pronounced seasonal community difference in the bacterial as compared to the viral communities. Procrustes superimposition (**c**) visualizes the coupling between viral and bacterial community similarities. The underlying ordinations are rescaled and rotated and paired samples are connected by a line. Gradually removing low abundant bacterial community members and Raup-Crick (presence–absence) based Procrustes analysis (**d**) suggests that abundant bacterial community members are important in shaping viral community composition.

Finally, we expected that the bacterial host community would have a strong structuring influence on the viral community. To achieve this, we sequenced 16S rRNA gene amplicons from these biofilm samples and used multivariate statistical tools to partition the variation in viral community composition explained by differences in environmental conditions and in host community composition. We first explored spatio-temporal patterns in viral community similarity, accounting for the relative abundance of vOTUs. In line with our previous analyses, unconstrained ordination revealed that viral communities clustered by season and, within each season, gradually changed with downstream distance and associated physicochemical parameter (Fig. 5a, SI Table 1). Constrained, distance-based ordination (db-RDA) indicated that these compositional changes were significantly explained by environmental variables, such as streamwater temperature, pH, electrical conductivity (a proxy for ion concentration), dissolved organic carbon and nutrient concentration. These environmental conditions change predictable with downstream distance and season, and hence, we assessed the variance in viral community composition explained by a reduced constrained ordination. Permutation indicated that downstream distance and season significantly (permutation test for db-RDA, *n* = 999, *p* < 0.01) explained viral community composition, however together they only explained 15.4% of variance in viral community composition. Biofilm bacterial communities also exhibited pronounced seasonal and longitudinal community turnover (db-RDA, explained variance: 41.3%). Using Procrustes rotation and permutation, we assessed the coupling between viral and bacterial community composition and found a significant coupling between these communities (symmetric Procrustes rotation correlation = 0.57, *p* = 0.001, Fig. 5b). There were no significant differences between Procrustes residuals among seasons (ANOVA, *F* = 1.93, *p* = 0.21), indicating that the coupling between viral and bacterial communities in stream biofilms is similarly strong throughout the year. Moreover, we explored whether the coupling between viral and bacterial community similarity may be driven by abundant bacterial community members. Assuming that phages in biofilms may also follow a killing-the-winner strategy, viral community composition may be coupled to the presence of abundant bacterial community members. To address this at a community level, we gradually removed rare bacterial OTUs and performed Procrustes tests between the viral community and the subsampled bacterial community. However, since abundance-based similarity indices are relative insensitive to the removal of rare taxa, we explored the coupling between the viral and bacterial community using Procrustes tests on the presence–absence based Raup-Crick similarity index. The presence-absence based coupling between viral and bacterial communities (symmetric Procrustes rotation correlation: 0.57, *p* = 0.001) was equally strong compared to the coupling considering relative abundances, indicating that presence and abundance of a bacterial host may be similarly relevant for the presence of viral community members. However, gradually removing low-abundance bacterial community members from the presence-absence matrix, we found that the coupling strength between viral and bacterial community composition was maintained to a large extend. Even bacterial communities subsampled to only 4% of the most abundant members (*n* = 57 OTUs) maintained significant correlation with viral communities. This is striking and indicates that, at a community level, viral communities appear to be linked to the presence of the most abundant bacterial community members. Taken together, these analyses illustrate that variance in stream biofilm viral community composition can be explained by spatiotemporal changes in the environment, but that the coupling to the bacterial community, particularly the abundant bacterial community members, explains more of this compositional turnover.

## Conclusions

In conclusion, we provide here the first glance at the community ecology of viruses in stream biofilms. We found a vast diversity of dsDNA viruses and show that these communities exhibit pronounced seasonal and longitudinal patterns. We provide the first evidence that the dispersal of viruses across distant stream biofilm patches may be facilitated by the presence of depolymerases coupled to a lytic viral life cycle, whereas lysogeny may be an alternative strategy to infect biofilm-dwelling bacteria locally. Finally, we demonstrate that much of the compositional turnover in the viral community can be deterministically explained by changes in bacterial community composition, and that changes in the presence and absence of abundant bacterial community members underlie this coupling. Given the role that viruses may play in shaping the diversity of all domains of life present in stream biofilms and the ramifications this may have on the functioning of stream ecosystems, further insights into the dynamics, infection and infection-avoidance strategies as well as the (co-)evolutionary consequence of viral predation are urgently needed.

## Supplementary information

Supporting Information
