# Supplementary material for: Viral diversity is linked to bacterial community composition in alpine stream biofilms
Source: ISME Commun. 2022 Mar 30;2:27. doi: 10.1038/s43705-022-00112-9 (PMC9723757; doi:10.1038/s43705-022-00112-9)
Supplement: Supplementary file 1 — Supporting Information [file 43705_2022_112_MOESM1_ESM.docx]

Supporting Information

**Spatiotemporal dynamics of viral diversity in alpine stream biofilms**

Bekliz M, Pramateftaki P, Battin TJ and Peter H

**SI Table 1** Key environmental parameter across sites and seasons.

**SI Table 2** Depolymerase protein IDs and corresponding database source (format: *database-protein_ID*) retrieved from Pires et al 2016, Knecht et al. 2020 and Latka et al 2017. See references in main text for more details on these viral depolymerases.

| rcsb_pdb-1DYO | uniprot-AAO47505.1 | uniprot-AFO10889 | uniprot-AGY46977 | uniprot-CBX45113 | uniprot-P5+NP_620343.1 | uniprot-YP_007003328 |
| --- | --- | --- | --- | --- | --- | --- |
| rcsb_pdb-1IB4 | uniprot-AAQ12204 | uniprot-AFQ96603 | uniprot-AGY46980 | uniprot-CBY99572 | uniprot-P7+NP_040700.1 | uniprot-YP_007003341 |
| rcsb_pdb-1K5C | uniprot-ABA54611 | uniprot-AFU63680 | uniprot-AGY47215 | uniprot-CBY99579 | uniprot-P7+NP_049902.1 | uniprot-YP_007007685 |
| rcsb_pdb-1RMG | uniprot-ABL61072 | uniprot-AFV51346 | uniprot-AGY47298 | uniprot-Gp16+NP_042004.1 | uniprot-Pb2+YP_006968.1 | uniprot-YP_007008117 |
| rcsb_pdb-2X3H | uniprot-ABQ88383 | uniprot-AFX93502 | uniprot-AGY47555 | uniprot-Gp16+NP_052116.1 | uniprot-YP+003714746 | uniprot-YP_007236804 |
| rcsb_pdb-3EQN | uniprot-ACE96035 | uniprot-AFX93505 | uniprot-AGY47556 | uniprot-Gp16+NP_523341.1 | uniprot-YP_001039683 | uniprot-YP_007237194 |
| rcsb_pdb-4CL2 | uniprot-ACH57080 | uniprot-AFX93507 | uniprot-AGY47760 | uniprot-Gp181+NP_803747.1 | uniprot-YP_001604152.1 | uniprot-YP_007348361 |
| rcsb_pdb-4MR0 | uniprot-ADA79896 | uniprot-AGB62649 | uniprot-AGY47935 | uniprot-Gp3+YP_002004530.1 | uniprot-YP_001604152 | uniprot-YP_007348539 |
| rcsb_pdb-4XOT | uniprot-ADA79897 | uniprot-AGC35227 | uniprot-AGY48030 | uniprot-Gp36+NP_853596.1 | uniprot-YP_002300374 | uniprot-YP_007348546 |
| rcsb_pdb-4XUO | uniprot-ADA82273 | uniprot-AGE60867 | uniprot-AGY48191 | uniprot-Gp36+NP_877475.1 | uniprot-YP_002332533.1 | uniprot-YP_007517417 |
| rcsb_pdb-5JS4 | uniprot-ADA82322 | uniprot-AGE60945 | uniprot-AGY48254 | uniprot-Gp4+NP_059632.1 | uniprot-YP_003347555 | uniprot-YP_007517493 |
| rcsb_pdb-5M5Z | uniprot-ADA82374 | uniprot-AGN12301 | uniprot-AGY48483 | uniprot-Gp5+NP_049757.1 | uniprot-YP_004286222 | uniprot-YP_007517572 |
| rcsb_pdb-5W5P | uniprot-ADA82474 | uniprot-AGO47740 | uniprot-AGY48730 | uniprot-HM214492 | uniprot-YP_004327331 | uniprot-YP_007517647 |
| rcsb_pdb-5W6H | uniprot-ADD80892 | uniprot-AGO48665 | uniprot-AHB81145 | uniprot-NP_046584.1 | uniprot-YP_004893855 | uniprot-YP_007673458 |
| rcsb_pdb-5W6S | uniprot-ADD80999 | uniprot-AGO49035 | uniprot-AHG23941 | uniprot-NP_073686.1 | uniprot-YP_005098420 | uniprot-YP_007675684 |
| rcsb_pdb-5ZRU | uniprot-ADF59152 | uniprot-AGO49248 | uniprot-AHN84645 | uniprot-NP_073695.1 | uniprot-YP_006383591 | uniprot-YP_007677121 |
| rcsb_pdb-6C72 | uniprot-ADR30483 | uniprot-AGO49328 | uniprot-AIA64067 | uniprot-NP_108727.1 | uniprot-YP_006488624 | uniprot-YP_007678034 |
| rcsb_pdb-6EU4 | uniprot-AEJ81510 | uniprot-AGO49696 | uniprot-CAJ29390 | uniprot-NP_112090 | uniprot-YP_006488654 | uniprot-YP_008050881 |
| rcsb_pdb-6NW9 | uniprot-AEW47853 | uniprot-AGO49697 | uniprot-CAJ29458 | uniprot-NP_112710.1 | uniprot-YP_006907302 | uniprot-yP_008051427 |
| rcsb_pdb-6TKU | uniprot-AEX65697 | uniprot-AGO49746 | uniprot-CBW38917 | uniprot-NP_690719 | uniprot-YP_006907824 | uniprot-YP_008060120 |
| uniparc-NP_690860 | uniprot-AEY69698 | uniprot-AGS80957 | uniprot-CBW38974 | uniprot-NP_690774 | uniprot-YP_006987059 | uniprot-YP_008060258 |
| uniparc-YP_004327544 | uniprot-AFH14728 | uniprot-AGY46593 | uniprot-CBW39031 | uniprot-NP_803297.1 | uniprot-YP_006987233 | uniprot-YP_008130362 |
| uniprot-006906201 | uniprot-AFM73722 | uniprot-AGY46829 | uniprot-CBW39181 | uniprot-NP_817333.1 | uniprot-YP_006987816 | uniprot-YP_008240873 |
| uniprot-AAA88489 | uniprot-AFM73788 | uniprot-AGY46940 | uniprot-CBW39235 | uniprot-NP_944291 | uniprot-YP_006990138 | uniprot-YP_008241124 |
| uniprot-AAL15086 | uniprot-AFO10349 | uniprot-AGY46975 | uniprot-CBX44510 | uniprot-P15+NP_040683.1 | uniprot-YP_007002901 | uniprot-YP_008242013 |
| uniprot-YP_950618 | uniprot-YP_654136.1 | uniprot-YP_214529 | uniprot-YP_009043945 | uniprot-YP_009036975 | uniprot-YP_009035268 | uniprot-YP_009007463 |
| uniprot-YP_950681 | uniprot-YP_654147 | uniprot-YP_240092 | uniprot-YP_009044242 | uniprot-YP_009042723 | uniprot-YP_009036150 | uniprot-YP_009010167 |
| uniprot-YP_007349243 | uniprot-YP_654148 | uniprot-YP_338127 | uniprot-YP_009056510 | uniprot-YP_009043178 | uniprot-YP_009036526 | uniprot-YP_009031349 |
| uniprot-YP_009003154 | uniprot-YP_009004869 | uniprot-YP_008430873 | uniprot-YP_008318292 | uniprot-YP_008242178 | uniprot-YP_008318429 |  |
